# Biosynthesis of Nanoparticles from Various Biological Sources and Its Biomedical Applications

**DOI:** 10.3390/molecules28114527

**Published:** 2023-06-02

**Authors:** Gopalu Karunakaran, Kattakgoundar Govindaraj Sudha, Saheb Ali, Eun-Bum Cho

**Affiliations:** 1 Institute for Applied Chemistry, Department of Fine Chemistry, Seoul National University of Science and Technology, 232 Gongneung-ro, Nowon-gu, Seoul 01811, Republic of Korea; 2 Department of Biotechnology, K. S. Rangasamy College of Arts and Science (Autonomous), Tiruchengode 637215, Tamil Nadu, India; 3 Department of Periodontics, Saveetha Dental College and Hospital, Saveetha Institute of Medical and Technical Sciences (SIMATS), Chennai 600077, Tamil Nadu, India

**Keywords:** biosynthesis, nanoparticles, antifungal, antibacterial, antidiabetic, biomedical applications

## Abstract

In the last few decades, the broad scope of nanomedicine has played an important role in the global healthcare industry. Biological acquisition methods to obtain nanoparticles (NPs) offer a low-cost, non-toxic, and environmentally friendly approach. This review shows recent data about several methods for procuring nanoparticles and an exhaustive elucidation of biological agents such as plants, algae, bacteria, fungi, actinomycete, and yeast. When compared to the physical, chemical, and biological approaches for obtaining nanoparticles, the biological approach has significant advantages such as non-toxicity and environmental friendliness, which support their significant use in therapeutic applications. The bio-mediated, procured nanoparticles not only help researchers but also manipulate particles to provide health and safety. In addition, we examined the significant biomedical applications of nanoparticles, such as antibacterial, antifungal, antiviral, anti-inflammatory, antidiabetic, antioxidant, and other medical applications. This review highlights the findings of current research on the bio-mediated acquisition of novel NPs and scrutinizes the various methods proposed to describe them. The bio-mediated synthesis of NPs from plant extracts has several advantages, including bioavailability, environmental friendliness, and low cost. Researchers have sequenced the analysis of the biochemical mechanisms and enzyme reactions of bio-mediated acquisition as well as the determination of the bioactive compounds mediated by nanoparticle acquisition. This review is primarily concerned with collating research from researchers from a variety of disciplines that frequently provides new clarifications to serious problems.

## 1. Introduction

### 1.1. History

The word “nano” was derived from the Greek word “nanos,” it means “little,” and it is the cognomen of the one-thousandth bit (109). Nanoparticles (NPs) confer a solid colloidal particle with at least one dimension ranging from 1 to 100 nm [1]. However, the majority of materials that are applied in drug delivery are in the range of 100–200 nm. Because of their unique electronic structure, massive conductivity, wide exterior position, and quantum size significance, these particles can change their chemical and physical properties. Nanoparticles are used in a variety of applications today, including anti-viral, cosmetics, electronics, and textiles [2,3]. Researchers have discovered numerous beneficial effects of nanotechnology, and it now plays an important role globally. Nanotechnology has created several material industries, including the food industry, and now its role in the biomedical field has increased, providing better results in the future medicine field [4,5].

### 1.2. Different Properties of Nanoparticles

The nanoparticles’ small size has several extraordinary properties that play an important role in various reactions [6]. Nanoparticles are nanoscale systems that are gaining popularity among researchers due to their ability to develop a wide range of shapes and sizes that can be used in a variety of advanced biotechnological applications. Nanoparticles have distinct physicochemical and optoelectronic properties that may be unique properties of implementations such as electronics, pharmaceutical compounds, chemical sensors, catalysts, antimicrobial agents, clinical diagnostics, and mapping. These features provide nanoparticles with more specific and significant effects than bulk particles or unique individual molecules. Due to these properties, improved electrical transport, stimulated roughness, and stability make it most effective for the medicine, textile, defense, agriculture, food, cosmetics, and space sectors [7]. Nanoparticles exhibit tremendous shape and size characteristics, as well as a broad spectrum of applications when compared to other bulk materials, and are used for broad-scope implementations [8].

### 1.3. Several Approaches for Nanoparticle Synthesis

**Figure 1:**
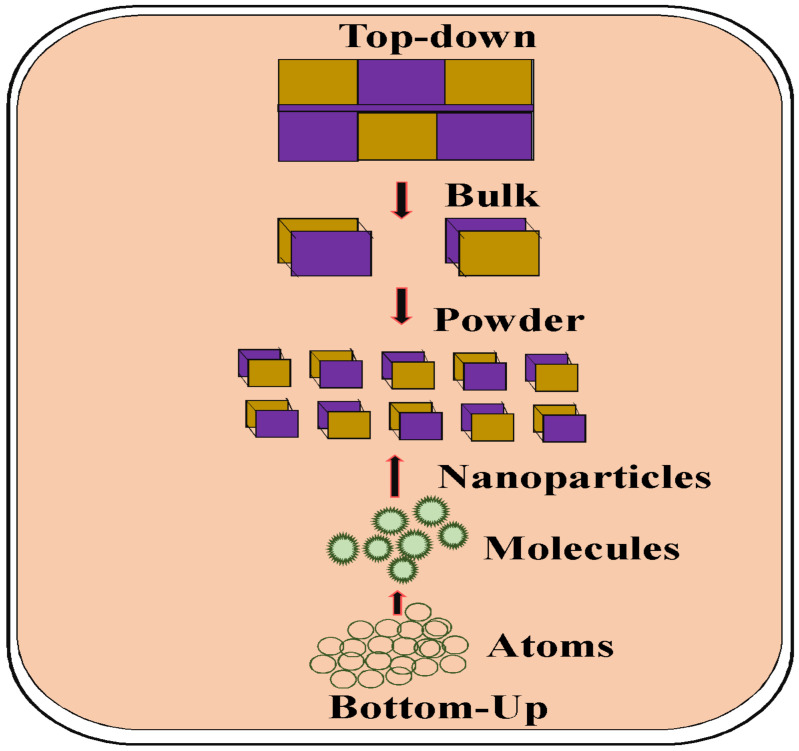
Schematic representation of top-down and bottom-up method for the obtaining of nanoparticles.

The top-down method, in which the synthesis begins with breaking bulk material into pieces and the development of nanoparticles, is the most widely used approach for nanoparticle synthesis. The bottom-up method involves gathering atoms and molecules to develop various nanoparticles. The most effective merit of the top-down method is that it facilitates the development of nanoparticles in large numbers in a short amount of time. However, the main merit of the bottom-up method is that it leads to the formation of nanoparticles with defined crystallographic properties and a higher specific surface area (Figure 1) [9].

A top-down approach for synthesizing the defined shape is not feasible. However, by using a bottom-up approach, waste components can be eliminated and fewer nanoparticles can be created. The chemical reduction method is one of the main approaches for obtaining nanoparticles using the bottom-up method. Commonly, the nanoparticle synthesis method might be categorized into three parts: chemical, physical, and bio-mediated approaches [10]. Additionally, this method was suggested as the most significant and widely used approach for synthesizing nanoparticles.

Temperature, pressure, and energy are all used in the physical approach to obtain NPs [11]. By the chemical method, NPs are obtained through sol–gel, atomic condensation, chemical etching, laser pyrolysis, spray-mediated pyrolysis, and sputtering. The morphologies of the nanoparticles can be altered by chemical and reaction ratios. After the synthesis, the obtained nanoparticles might encounter difficulties in terms of bioaccumulation, the toxic nature, regrowth, reuse, and recycling [12,13]. However, green-approach-mediated synthesized NPs have proven to be non-toxic [14].

The biosynthesis of nanoparticles with distinct sizes and shapes has been used for a variety of applications in biomaterials science [15,16]. Nanoparticles have been developed for several applications in the pharmaceutical field to treat several viral and bacterial diseases [4]. The biosynthesis approach has various merits compared to other classical synthesis protocols because of the capacity for bulk production under eco-friendly approaches. The vast biological diversity and easy sources of plant output have been thoroughly investigated for the amalgamation of nanomaterials. These biologically synthesized nanomaterials have significant applications in various fields such as diagnosis, treatment, manipulation of surgical nanodevices, and other product formations. Nanomedicine has shown promising clinical results in the management of diverse chronic diseases. Moreover, eco-friendly methods of obtaining NPs were marked as fundamental materials for the upcoming generations to protect from several diseases [1].

### 1.4. Antibacterial and Antiviral Properties, the Importance of Zeta Potential, and Mode of Action of Nanoparticles

Greenly synthesized metal and metal oxide NPs such as Au, Ag, Pt, ZnO, and Se NPs are used in pharmaceutical products, cosmetics, antimicrobial applications, and medical applications. Bio-manipulated nanoparticles are currently being used in clinical settings for the diagnosis, treatment, transportation, and manipulation of specific medicines [3]. Metallic NPs such as Ag, Au, Zn, Pt, Fe, Ni, Cu, Mg, Ti, and their oxides have provided clear ideas of the process of obtaining them for various decades [4]. The variously obtained nanoparticles show several uses in different fields, such as chemical sensing, optical elements, catalysis, pharmaceutical agents, and antibacterial agents.

Silver nanoparticles (AgNPs) are highly reactive and are coupled with tissue proteins, causing morphology changes in the bacterial nuclear and cell membranes, resulting in cell breakage and mortality [17]. The destruction of the cell wall takes place when the nanoparticles come into contact with the cell wall. Maillard et al., gave a detailed explanation of the role of the zeta potential in antibacterial activity [18]. According to this review, the zeta potential is a very useful tool to understand the viability and membrane permeability of bacteria and how the zeta potential of materials is important to develop new advanced antibacterial materials. In a study, ZnO nanoparticles were used to evaluate their antimicrobial properties using different zeta potential bacteria from −14.7 to −23.6 mV. In this study, it was shown that Gram-positive bacteria have a lower zeta potential value, whereas Gram-negative bacteria have a higher zeta potential value [19]. In this study, the ZnO zeta potential value was altered to see the effect on different bacteria. An attempt was made to neutralize the opposite zeta potential, which is the positive and negative value, and to thus have a higher positive charge. ZnO nanoparticles had a higher antibacterial activity against negatively charged Gram-negative bacteria. Thus, this study confirms that the zeta potential plays an important role in the design of antibacterial agents. Ag can also interact with bacterial RNA and DNA by damaging them and suppressing bacterial growth. Ag has stronger antifungal and antiviral properties. Ag metal and Ag coatings are applied in considerable amounts and have no side-effects in the human system against various diseases, due to viruses, bacteria, fungi, and yeast [3].

AgNPs exhibit better efficiency toward bacteria and less toxicity to the human system. Commonly, Ag ions might be attached to negative-power molecules such as DNA, RNA, and proteins. Silver NPs, obtained through biological methods, exhibit anti-bacterial activity toward bacteria, including the methicillin-resistant *Staphylococcus aureus* (*S. aureus*) [4].

Yazdanian and coauthors showed that AuNPs were also found to be an excellent antibacterial obtained from *Alternanthera philoxeroides*-mediated green synthesis. The obtained nanoparticles were around 72 nm in size [3]. CuNP was synthesized using *Cardiospermum halicacabum* as a biosynthesis, and the synthesized nanoparticles were 30–40 nm in size with a hexagonal shape. The obtained CuNP was found to inhibit the bacterial cell wall and inhibit the growth of bacteria [3]. FeNPs were also obtained using *Euphorbia hirta* through green synthesis, and the obtained nanoparticles were 25–80 nm in size with a cavity-like structure. They showed excellent antibacterial and antifungal activities against various pathogens [3]. TiNP obtained using *Azadirachta indica* was found to be around 18 nm with crystalline structures and to possess outstanding antibacterial activity against various bacteria [3]. Further, ZnONP obtained using *Costus pictus* was found to be around 40 nm in size and have excellent antibacterial and antifungal activities [3].

Viruses are a major threat to humans, as about 60% of major illnesses are due to virus-based infections [20]. The virus infects through respiratory or enteric channels. Viruses are transferred in different ways, such as by surface contact, direct transfer from an infected person to another person through secretions, aerosol particulates, and inanimate surfaces [21,22,23,24]. There is a demand for the development of antiviral agents to control infections caused by viruses [21,22,23,24]. According to Choudhary et al. (2020), tiny AgNPs are acceptable to human immune viruses, and the coupling of Ag nanoparticles of 5 nm with the gp120 protein of the HIV suppresses the virus by targeting itself in the host tissue [22]. Gold nanoparticles of around 17 nm were also inhibiting HIV-1 infections through fusion [21]. 

Copper iodide nanoparticles of around 160 nm were found to inhibit the *Feline calicivirus* infection by ROS generation and also by the oxidation of capsid proteins [21]. Cuprous oxide nanoparticles of around 45 nm were found to inhibit the *Hepatitis C* virus infection by preventing the HCV virus’s entry through attachment mechanisms [21]. Iron oxide nanoparticles of 10–15 nm were also found to be very effective in inhibiting the H1N1 influenza virus infection by inactivating the cellular proteins by interacting with the proposed -SH functional group [21]. ZnO nanoparticles of 16 to 20 nm were also found to be effective against H1N1 influenza, which inhibits the proliferation of the virus [21]. Up until now, various metal nanoparticles have been obtained and analyzed for their different biomedical applications. Hence, in this review, we describe the various synthesis methods for NPs and their biological mechanisms to feature their various biomedical applications.

## 2. Different Types of Nanoparticles

**Figure 2:**
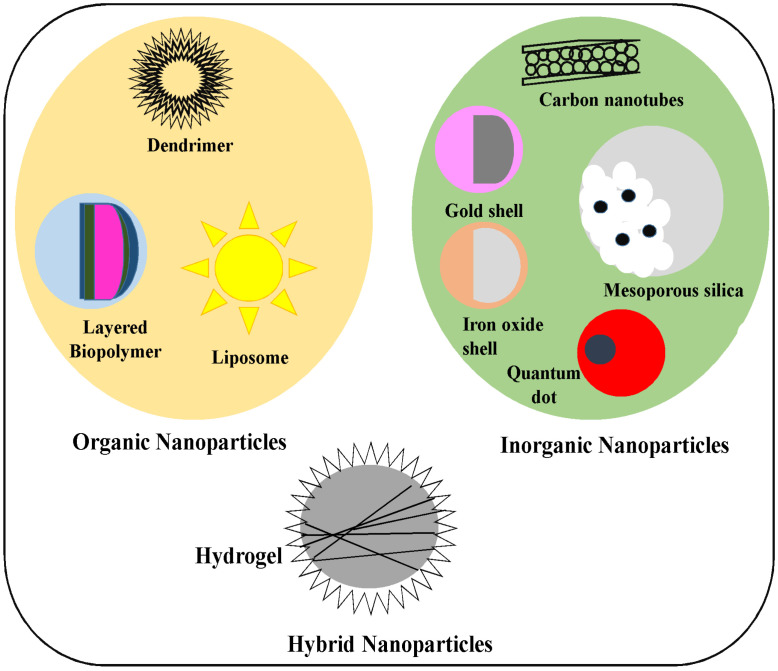
Schematic representation of various types of nanoparticles.

Generally, nanoparticles are categorized into three kinds: organic (polymers, proteins, and lipids), inorganic (salts and metals), and hybrid (nanofoams) (Figure 2). Organic nanoparticles or polymers were identified as dendrimers, micelles, liposomes, and ferritin. These particles are not toxic or biocatalytic, and a few particles such as micelles and liposomes include a hollow core, which is marked as a nanocapsule, and are sensitive to electromagnetic rays such as light and heat [25]. Numerous organic chemicals are used in different pharmaceutical products, such as dyes, flavors, inks, and household products. In several of these products, the organic chemicals are diluted for development or chemically altered to enhance the particle properties. Chemicals are not dispersed in a liquid that is essential for development; their properties and applications are effectively restricted [26].

The pharmaceutical outcome has limitations in bioavailability and efficiency because it is not soluble in water. This might be due to limitations in the formation of new drugs or the area of recent medicine [27]. The same can be said for nutraceuticals, biocides, and a variety of other important essential compounds. For developing a tiny dilution of organic components, non-soluble particles might be used to act as purely dispersed molecules. It is not essential to develop new chemicals or utilize burnable, toxic, or vaporous solutions. This selection was necessary for the development and formation of new results due to the extent of chemical components that must be accessible, which gives high importance to a novel and merciless outline. Organic nanoparticles may provide a solid material coupled with organic components such as polymers or lipids, particularly at scales ranging from 10 nm to 1 m. The particles attained more intention as compared to inorganic particles, where numerous studies and trade speculations were being formed [28].

In recent years, the pharmaceutical industry has conducted interesting research on organic nanoparticles, as a result of which nanomedicine has enabled the formation of new particles and the purification of better-developed methods. The formation of dendrimers, protein connections, DNA-transferring devices, liposomes, and shell-over-coupled brick co-polymer micelles may be noted for nanoparticles synthesized from the “bottom-up” method of transferring active molecules. A “top-down” approach has gained more researcher interest, as have methods such as wet nano-milling to grind large materials and attain material separation with sub-micron maximum material cadence. Various wet methods to develop colloidal dilutions form liquid mixtures, which critically depend on the particular excretion of the oil phase of an amalgam and ensure solidification through precipitation, enclosure, and crystallization of any organic particles that are dispersed within the solvents dropwise [29].

All of these techniques have been proven to be purely generic, and they may have been limited to specific classes of particles classified by their chemical reactivity or physical characteristics. Organic suspension environments, which might be diluted by dropwise differentiation from their inorganic constituents, do not hold organic nanoparticles tenaciously in the environment for a lengthy manufacturing process [30].

Organic and inorganic hybrid nanoparticles are most effective at attracting people’s attention. Combining inorganic and organic substances in the nanoscopic range is not a novel idea. Nature has a plethora of hybrid particles such as nacre, bone, and corals that interact with biogenic substances and inorganic agents to transfer hybrid biomaterials for the benefit of humans [31]. Inorganic and organic combined nanomaterials are used in various fields, including optics, optoelectronics, coating, and bio-clinical applications. In common terms, polymer substances have critical structural properties that can be manipulated for their mechanical properties and accessibility at the end products, whereas the inorganic substances might induce unique properties such as luminescence, magnetism, and catalytic features to induce the heating and mechanical features in the organic materials [32].

Metal and metal-based nanoparticles are frequently classified as inorganic nanomaterials. Nanoparticles have various advantages, such as their small size and higher surface area, which have stimulated the interest of researchers in recent years, and they are now being used in a variety of biological and engineering fields [33]. In comparison to organic and hybrid nanoparticles, inorganic nanoparticles are now modified with several chemical features that allow them to bind with antibodies, drugs, and ligands, launching a different range of significant implementations in filtration, drug delivery, biotechnology, and as carriers for gene-mediated transfer with improved disease mapping [34]. However, this mapping has developed in recent years as magnetic mapping, computed tomography, ultrasound, and Raman spectroscopy have been used to map several disease stages. Nanoparticles such as gold, titanium, and silver are developed for use in numerous mapping applications. Furthermore, nanoshells and nanocapsules have been created to use mapping methods in a broad implementation. In the past few decades, nanoparticles such as Au, Ag, and magnetic nanoparticles have been effectively utilized for their use in disease treatment and as diagnostic agents. The future usage of inorganic nanoparticles such as silicas, quantum dots, titania, and many catalysts is not an inquiry but will give nano-solutions a more fanciful time in the numerous non-dispersible or imperfectly soluble organic components that are applied over various improved technologies and materials outlined [35].

Various types of inorganic nanomaterials and various methods of obtaining them have made it possible to manipulate new drug delivery systems. Several significant issues should be considered before converting these inorganic nanomaterials into medicinal products. Differentiated from the better-organized organic nanoparticles, the medicinal conversion of inorganic nanomaterials for drug delivery is still under stable argument because of the absence of a stronger proof and data relating to biological safety, especially the bio-catalytic features, elimination methods, and long-use toxicity assays to support its in vitro and in vivo biological safety. The biocompatible inorganic particle-dependent nanomaterials provide an unrivaled opportunity and demonstrate a better scope for effective clinical implementation in a variety of diseases [36].

## 3. Various Biosources Are Used to Synthesize Nanoparticles

### 3.1. Green Synthesis of Nanoparticles

Green-mediated nanoparticle synthesis is a low-cost, environmentally friendly method with no toxic properties. This method uses various stabilizing and reducing substances, such as plants, microbes, and some natural agents, to develop NPs. The green-mediated obtaining of nanoparticles has gained popularity due to their low cost, non-toxicity, and high stability. The green-mediated synthesizing approach was an eco-friendly method to manipulate nanoparticles that did not cause toxic effects on the environment or human health. The conventional approach might manipulate nanoparticles in large amounts with defined shapes and sizes. Furthermore, these approaches necessitate massive economies, are difficult, and adhere to outdated protocols. The green synthesis method has numerous advantages over chemical and physical methods, including ease of development, simplicity, low cost, and low waste in the development of NPs [37,38].

**Figure 3:**
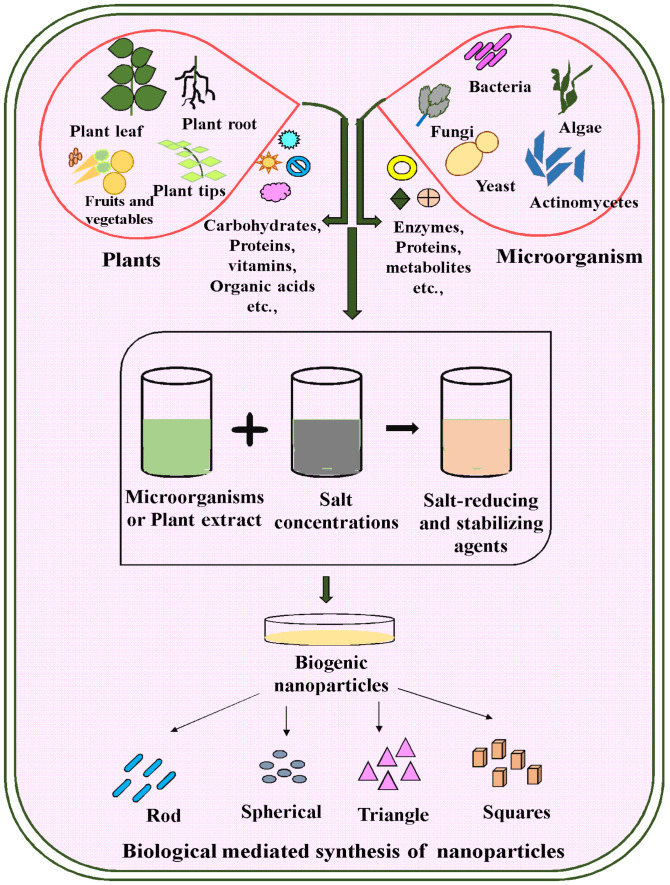
Pictorial presentation of biologically mediated synthesis of nanoparticles.

The green-mediated approach to obtaining NPs is differentiated, but organisms or their extracts are easily responsive to metallic salt, and biological stabilization was analyzed to turn metal into NPs. The formation of nanoparticles by organisms was a green method that used fungi, bacteria, and viruses’ enzymes and secondary metabolites. These kinds of organisms provide primary substances for the synthesis and manipulation of better-organized nanoparticles [39]. For differentiation, the microbially mediated obtaining method was a significant one, and plants might be applied as a beneficial method for nanoparticle formation. For obtaining nanoparticles, it might be easier to use plant extracts. Furthermore, plant extracts may reduce metallic ions, allowing microbes to easily form and stabilize metallic nanoparticles [40,41]. Plant extracts and various components such as proteins, polysaccharides, amino acids, and phytochemicals such as flavonoids, alkaloids, tannins, and polyphenols are appearing that might stabilize and reduce the nanoparticles (Figure 3).

### 3.2. Plant-Based Synthesis of Nanoparticles

Plant parts such as leaves, stems, flowers, bark, roots, fruits, vegetables, and shoots are used as a primary compound for the synthesis process of nanoparticles. A variety of plant leaf extracts are used for synthesis, which includes bioactive components that occur during the synthesis process using an eco-friendly approach [42,43]. Plant extracts are used to obtain metallic nanoparticles, but recent research has demonstrated an effective and unique method for obtaining nanoparticles via well-managed purified plant components [44,45]. Plant bioactive components are used as effective cancer drugs to treat a variety of cancers and dangerous diseases (Table 1).

As a result, the downstream processing-related scale-up approach effectively stimulated and reduced manipulation duration. Many studies have suggested the use of extracted phenolic components such as secondary metabolites, sugars, and proteins for the synthesis of various metallic nanoparticles. Plants or their extracts, particularly flavonoids, have been used for the bioremediation of metals for a lengthy period. Plant metabolite flavonoids, such as quercetin, were applied to biosynthesize copper and silver nanoparticles in micelle suspension, which have better antibacterial activity and a higher surface area. The *Desmodium triflorum* plants were given silver ions in an agar suspension, followed by shooting, resulting in an increase in the concentration of silver ions in the tissue, and this clearly shows the uptake of silver ions by plants [46]. The detoxification, capping of silver, and development of AgNPs in the tissue were observed. The different nanoparticles developed with *Chrysophyllum oliviforme* plants are in the range from 20 to 50 nm in diameter and have less polydispersity, which reveals the effective in vivo synthesis of nanoparticles. These materials allow for the development of a higher positioning of materials, with the possibility of synthesis [47]. The extract of *Veronica amygdalina* was observed for the bioconversion of silver ions. Faster development of silver nanoparticles with a merit size of 15 nm was achieved, as was a controlled scale of material size between 2 and 18 nm. Terpenoids such as geraniol, linalool, and citronellal found in geranium leaf extracts are feasible reducing substances for silver ions [48].

### 3.3. Amalgamation of Nanoparticles Using Marine Algae

For the amalgamation of marine algae nanoparticles, different literature data are available (Table 2). The brown seaweed *Sargassum wightii* was stated to have the ability to synthesize nanoparticles with a defined range between 8 and 12 nm [62]. The synthesis of nanoparticles using seaweed (*Fucus vesiculosus*) is reported to have the capacity for Au bio-absorption and bio-reduction reactions, which are useful to extract Au from dispersing hydrometallurgical solutions and electronic scrapes for obtaining NPs with different shapes and sizes (Figure 3). The synthesized nanoparticles made from algae extracts produce that stabilizing effect on cotton fabrics with excellent antibacterial activity. *G. acerosa* was stated to include the significance of obtaining antifungal Ag NPs [62,63].

### 3.4. Bacteria-Mediated Synthesis of Nanoparticles

Multicellular and single-cellular organisms are used to produce materials that might be intracellular or extracellular. The bacterial enzymes occur through an intracellular signaling mechanism for the conversion of ions into NPs [67]. The main merits of the bacterially mediated amalgamation of nanoparticles are that it is easy to use and better controls the whole process of biosynthesis. Following genetic engineering methods, the development could be formed quickly for effective purposes such as reducing toxic effects and achieving defined nanoparticle synthesis [68]. This method also has some restrictions, such as downstream reactions, a cost-effective but laborious approach, and the need to maintain control over size and shape.

Moreover, metal nanoparticles are synthesized through intra- and extracellular bacterial extracts. The extraction process of nanoparticles uses mainly cellular extract via the downstream method. In this protocol, a diverse group of sulfate-reducing bacteria, such as *Acinetobacter calcoaceticus* and *Desulfovibrio desulfuricans,* is used for the controlled development of nanoparticles of controlled size (Figure 3 and Table 3). These bacteria have a unique feature that gives them the capability to convert precursor solutions into nanoparticles. At 37 °C and a neutral pH, the reducing bacterium *Rhodopseudomonas capsulate* was used to obtain nanoparticles by using elemental nanoparticles [69].

### 3.5. Fungi-Mediated Synthesis of Nanoparticles

Various fungal species are used to obtain different NPs (Table 4). The obtained nanoparticles, which include various microorganisms and fungi, are more effective prokaryotes. Fungi have some advantages over other microbial methods of obtaining nanoparticles in that it is easy to develop media, scale-up formation is easier, downstream reactions are easier, an increased number of proteins are generated, and biomass is simple to prepare. However, fungal enzymes increase the number of nanoparticles synthesized and have achieved the reductive properties required for the production of stable nanoparticles. Commonly, extracellularly produced nanoparticles are low in toxicity. Nanoparticles are developed extracellularly at 37 °C with a wide range from 15 to 30 nm via *Fusarium oxysporum* [79]. Moreover, this research provides that the obtaining of nanoparticles by fungal extracts would be through the processes of reduction and stabilization of substances [80].

The synthesis of fungal-associated nanoparticles is a hot topic in the scientific community. Fungi have been commonly used for the biosynthesis of nanoparticles and have attracted researchers’ attention for their ability to develop well-managed shapes and sizes of nanoparticles [88]. Fungi have been commonly used for the bio-mediated obtaining of nanoparticles, and better-managed dimensions might be obtained. Fungi, as they differentiate from bacteria, produce a higher ratio of NPs. Fungi excrete increased protein ratios, which effectively alter the formation of nanoparticles [89].

### 3.6. Actinomycetes-Mediated Synthesis of Nanoparticles

Actinomycetes have significant features similar to fungi and prokaryotes such as bacteria. It was concluded that the Ag and Au NPs are stabilized by proteins. Free amino acids or cysteine remain on proteins that have been coupled with Au NPs. Initially, gel electrophoresis reveals that the actinomycetes excrete four particular proteins of molecular aggregate in the range from 10 to 80 kDa. Hence, it was confirmed that the reduction and stabilization of the gold nanoparticles were brought about by various proteins present in them [90].

Thus, by developing various proteins and their ability to connect with various crystallographic features of gold nanocrystals, it is possible to develop complex shapes with manageable sizes. *Actinomycetes* have unique properties for which secondary metabolites can be used to create antibiotics. It was noted that *actinomycetes* play a significant role in the synthesis of metal nanoparticles [91]. An alkalothermophilic actinomycete obtained Au ions of 8 nm in size under extremely alkaline conditions and at constant temperatures. *Rhodococcus* sp. and *Thermomonospora* sp., both alkalotolerant actinomycetes, are used to produce gold nanoparticles. Intracellularly synthesized gold nanoparticles with diameters of 5 and 15 nm were also created. According to the findings, the ratio of NPs on the cell membrane was higher than on the cell barrier. The enzyme formed on the cell barrier and cell membrane was responsible for the formation of Au NPs. The cells were followed to develop nanoparticles, which revealed that the obtained nanoparticles were non-toxic to the cells [91].

### 3.7. Yeast-Mediated Synthesis of Nanoparticles

Biological reactions can manage the shape of materials better. In the log phase, *Schizosaccharomyces pombe* synthesized semiconductor nanocrystals. Extracellularly synthesized particles from microorganisms have a broad spectrum of benefits because protein-mediated interactions with the organisms make it easier for downstream reactions (Figure 3 and Table 5). Yeasts, a group of ascomycetes in fungi, have been shown to have more significant capabilities for obtaining NPs. Au NPs have been obtained intracellularly through the fungus Verticillium luteoalbum. The ratio of material development and the scale range of the NPs could be areas to explore by controlling physical parameters such as pH and temperature.

The tremendous amount of published data showed that the entire yeast family might aggregate various heavy metals. They can aggregate potential ratios of increased toxic metals. Several reports concluded that extracellular polysaccharides or peptides, which manage the cell barrier of heavy metals against or to active efflux from the cell, are the various mechanisms formed through these species that overwhelm the toxic properties of heavy metals [92]. Direct administration of intracellular metal ions required yeast cells to eliminate negative or fetal outcomes. Toxicity to cells is reduced by maximizing metal ion retention or by exposing cells to metals that do not have the same potential properties as lead, mercury, and cadmium.

**Table 5:** Yeast-mediated synthesis of nanoparticles.

Yeast	Type of Nanoparticles	Size of Nanoparticles	Biological Activities	Reference
*Saccharomyces* *cerevisiae*	Selenium	30–100 nm	Antimicrobial	[93]
*Saccharomyces* *cerevisiae*	Silver	100 nm	Antibacterial	[94]
*Saccharomyces* *cerevisiae*	Palladium	10–100 nm	Photocatalytic activity	[95]

The greater difference in size, particle arrangement, mono-dilution, and features is due to various mechanisms studied using yeast strains from different families for nanoparticle formation. GSH (glutathione) and two classes of metal-accepting coupling metallothioneins and phytochelatins (PC) were used to create a detoxification mechanism in yeast cells. In the majority of the yeast species reported, these molecules analyzed the pathway for the development of NPs and stabilized the compounds. Resistance was defined as the ability of yeast cells to change the absorption of metal ions into non-toxic compound polymer complexes. The yeast is commonly marked as “semi-transfer crystals” or “quantum semi-transfer crystals.” Current research has revealed that yeasts can also form other nanoparticles. In eukaryotes, yeast species are the most studied for bioprocesses [92].

Extracellular methods were used to obtain AgNPs from *Cladosporium cladosporioides.* It was stated that the proteins, organic acids, and polysaccharides excreted through fungus were capable of comparing various shapes and were used to regulate the formation of spherical crystals. *Penicillium fellutanum* was collected from coastal mangrove sediment, and using this sediment, AgNPs were synthesized by the extracellular synthesis method [92].

## 4. Biomedical Applications of Nanoparticles

### 4.1. Antibacterial Activity

**Figure 4:**
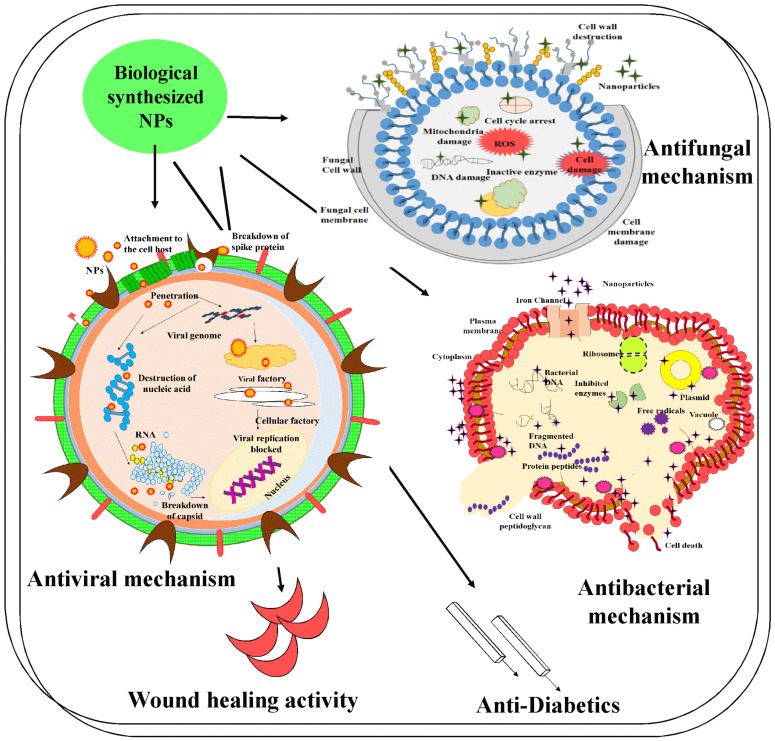
Application of biologically mediated synthesized nanoparticles in biomedical field.

In disease-causing organisms, nanoparticles are designed to break the polymer sub-group of the cell membrane. The opposite role of NPs effectively disrupts protein formation and damages cell membranes in bacterial cells. Silver nanoparticles at higher concentrations induced membrane rupture compared with low concentrations and effectively damaged the bacterial cell wall (Figure 4). *R. apiculate*-mediated silver nanoparticles showed a lower rate of growth than silver-nitrate-exposed bacterial cells, which could be because of particle size and increased exterior interaction, which resulted in induced cell membrane rupture and cell interruption [96].

The current study reported on the significance of Pt nanoparticles in human health and their application to various disease targets via microbes. Microbes show effective resistance to many antibiotics [97]. However, the formation of nanoparticles along with strong antimicrobial activity was effective in the biomedical area. Several studies on metallic nanoparticles such as Ag, Au, Pt, Pd, ZnO, and TiO_2_ have depicted a strong role in their antibacterial properties toward pathogenic microbes. Several metallic NPs, including Au, Ag, Pt, and ZnO, have strong cell death mechanisms. Another major feature of nanoparticles is their effective negative zeta potential, which induces antibacterial activity [98].

Apigenin from chamomile extract was used to create Pt nanoparticles with effective antibacterial potential against *S. aureus.* Previously, researchers discovered that the development of *E. coli* is suppressed by Pt electrolysis [99]. Another study found that a combination of Pt nanoparticles and partial ammonium had antibacterial effects by significantly suppressing Streptococcus mutans. Pt mixtures of polyamide S, including sulfones, reveal stronger antibacterial properties against *E. coli* and *S. aureus* [100].

Silver-Pt nanoparticles doped with particles of a size scale between 2 and 3 nm have induced significant antibacterial properties against *K. pneumonia*, *P. aeruginosa*, *E. coli,* and *S. choleraesuis*. Recent research has stated that the suppression of bacterial development is due to ATP generation and mitochondrial membrane stability. In addition, other reports stated that the implementation of polyvinylpyrrolidone-doped Pt nanoparticles with unique shapes and size scales ranging from 2 to 20 nm toward *P. aeruginosa* analyzed their antibacterial properties [101]. PVP-coupled Pt nanoparticles with diameters of 5.7 and 5.8 nm were exposed to *S. aureus* and *E. coli* Gram-negative bacteria, which are nanoparticles with small sizes that effectively suppress *E. coli* growth. Pt and Ag mixed nanoparticles are used for the reduction of graphene oxide (rGO) nanosheets, along with holes that lead to the induction of antibacterial properties against *E. coli* in the membrane between metal components, the rGO matrix, and bacteria. When combined with Pt NPs as a nano-mixture, polyvinylpyrrolidone (PVP) exhibits effective antibacterial properties against *K. pneumonia*, *Lactococcus lactis,* and *E. coli* [102]. Krishnaraj et al. (2010) stated that the *A. indica*-mediated Ag NPs strongly deplete the water-related pathogenic bacteria at a low concentration [103].

Based on the epidemic of infectious diseases caused by various pathogenic bacteria, there is an emerging need to discover novel antibacterial materials [104,105]. Several classes of NPs, such as Mg, Ti, Cu, or alginate, have effective antibacterial properties; gold and silver NPs have demonstrated a strong antiviral, antibacterial, and antifungal effect. The broad range of antibacterial properties of metallic nanoparticles, primarily gold and silver, encourages their use as disinfectants in the sterilization process of common water, medicine, food preparation, makeup, and various domestic items [106].

The biosynthesis of nanoparticles with antibacterial properties from fungi, bacteria, and algae to plant and tree root, leaf, bark, and tuber extract, and the mycosynthesis of Ag nanoparticles have excellent antibacterial activity against a variety of human-infection-causing organisms, including multidrug-resistant *S. aureus* and *S. epidermidis.* Similarly, the fungal strain *Aspergillus* was implanted for the extracellular synthesis of strong silver NPs with antibacterial properties against methicillin-resistant *S. aureus* and *S. epidermidis* [107]. Silver nanoparticles bio-mediated by *Aspergillus oryzae* filamentous mold showed antibacterial properties against *S. aureus* KCCM 12256. *Bipolaris nodulosa* fungal species might act as stabilizing substances for silver nitrate, which leads to the formation of silver nanoparticles in *P. vulgaris* and *B. subtilis* pathogens [108]. Silver nanoparticles were bio-mediated and obtained via gilled mushrooms of the species *Pleurotus sajor-caju,* which were effective against *S. aureus* [109].

*Phoma glomerata fungal* plant pathogens were studied for the production of silver nanoparticles and better antibacterial potential against *S. aureus. Trichoderma viride*-mold-species-bio-mediated vancomycin binds to nanoparticles and has demonstrated properties against vancomycin-resistant *E. coli.* Metal-reducing *S. oneidensis* was implemented for the biofabrication of Ag nanocrystals. Bacterial toxicity assays revealed that the obtained biogenic silver NPs had better bacterial properties toward *S. oneidensis* than other methods of obtaining colloidal silver NPs [110].

Several marine algae species are used to improve the antimicrobial properties of bio-mediated silver nanoparticles against human infectious pathogens *P. vulgaricus*, *Klebsiella* sp., *E. coli*, and *P. aeruginosa*. *Garcinia mangostana* leaf extract was tested in silver nanoparticles for biofabrication. For significant results, antibacterial assessment of human pathogens *S. aureus* and *E. coli* is performed using standard disc diffusion. The biosynthesis of silver nanoparticles and their properties toward the bacterial pathogen *V. cholera* are also assessed. While antibacterial agents are used, changes in membrane porousness and the reciprocal silver NP exposure in bacterial cells have been reported [111].

Antibacterial activities of Ag nanoparticles interacting with sodium alginate films are exposed to *S. aureus* strains, and disc diffusion depicts an antibacterial potential toward bacteria. The antibacterial coating is implemented on the pear and carrot exteriors, and the outcome is differentiating between exposed and unexposed samples. The silver nanoparticles significantly break the polymer subgroups of the cell wall in disease-causing bacteria. The considerable role of NPs frequently damages the cell barrier and breaks protein pathways in bacteria. The higher ratios of Ag nanoparticles cause quicker membrane damage than the low ratios and significantly damage the cell membrane of bacteria.

The *Rhizophora apiculata*-stabilized Ag nanoparticles reveal a lower ratio of bacterial growth on the culture plate compared to silver-nitrate-exposed cells; this might be because of the lower size of the materials and higher exterior interaction, which results in increased membrane damage and cell breakage in bacterial cells [112].

### 4.2. Fungicidal Activity

General antifungal agents can generate several side-effects such as diarrhea, increased renal failure, nausea, and increased body temperature; hence, for fungal diseases, substitutive treatment is needed. A recent study stated that Pt nanoparticles exhibited antifungal activity against several hazardous fungi such as *C. acutatum*, *D. bryoniae*, *C. fulvum*, *P. capsici,* and *P. drechsleri*. Biopolymer-based Pt nanocomposites GKPt NPs are tested for antifungal activity against various strains of fungi, such as *A. parasiticus* and *A. flavus.* Prior research has stated that the antifungal properties of Pt nanoparticles in a nano-mixture-induced membrane breakage raised the ratio of ROS, changed the shape of the mycelia, and resulted in damage to DNA and cellular breakage (Figure 4) [113].

The fungicidal activity mechanism of biosynthesized metallic NPs has a higher efficiency compared to commonly available antibiotics such as amphotericin and fluconazole. The plant-extract-procured silver NPs have effectively revealed membrane breakage in *Candida* sp., which interrupts fungal intracellular constituents and results in cellular damage [114]. Some of the available antifungal substances have restricted implementation and also minimized activities and the lack of curing of microbial infections. The wide-spectrum properties of silver nanoparticles have an interesting activity toward spore-spreading fungus and significantly damage fungal development. Exposure to NPs effectively altered the fungal cell wall structure.

### 4.3. Anti-Plasmodial Activity

Recently, the main factors that generate disease are spreading ubiquitously through trajectories. Trajectory management is a crucial need in the current state. Further, the developed anti-plasmodial species’ unique management method has a higher cost and lower efficiency to manage the specific organism in the medical field. However, efficient and predominant antimalarial medicines remain needed to manage plasmodial properties. In previous years, plants were used for customary agents of usual outcome and had all the substances for drug manufacturing for antimalarial disease. Secondary phytochemical components such as artemisinin and quinine constituents have been effectively used against the resistant malaria parasite. The substitute drug was required for managing the various strains based on increased parasite defense. The plant extract produced metallic NPs such as Ag, Pt, and Pd NPs, which help control malarial growth. The bio-mediated amalgamation of metallic Ag NPs by plant extract has halted malarial growth [115].

### 4.4. Antiviral Activity

Antivirals have been studied in conjunction with different degrees of completion for Hepatitis C, and target-acting drugs have resulted in a higher 90% treatment ratio. Despite the better outcome revealed with Hepatitis C, target-acting antiviral technology was not followed, to induce activity due to limitations in availability [116]. Several viral infections, including specific virus targets, have been left unfinished due to the urgent need for antiviral drug resistance. Current research has found a link between pharmaceuticals and a specific viral target. Furthermore, developing technologies that interact with altered host components can induce a viral disease cure. One feasible target was host cell agents that are needed for viral duplication but are excreted by the host. These specificities reduce viral growth via termination duplication and also lower the activities of the host [117]. The inherent viral infections were the first line of defense against viral dysfunction and death. The secondary host was targeted to induce the immune action that led to tissue damage in the reaction of viral elimination. The host immune response may be another viable target for infection cure, to induce host-mediated viral control while restricting tissue-breaking immunopathology. Furthermore, antiviral therapy may stimulate collaboration with target-acting antivirals with the goal of hosting immuno-manipulation to clear up both anguish and death etiologies.

Plant-associated NPs serve as replacement drugs in the treatment and management of viral diseases. The virus’s entry into the host is extremely dangerous, and it requires a faster adaptation reaction to develop its growth. The biosynthesis of silver nanoparticles might play an efficient role as wide-scope antiviral substances to limit virus cell features. Ambrose et al. (2022) stated that the bio-mediated synthesized silver nanoparticles have effective anti-HIV agents at the prior step of the backward transcription mechanism [118]. Nanomaterials have effective antiviral substances that suppress the virus before it enters the host system. The bio-mediated synthesized metallic NPs have various coupling actions to enable them to interact with groups of viral cells to manage the features of viruses. The bio-associated NPs play a strong broad-spectrum agent role against cell-free viruses and cell-mediated viruses. Furthermore, Ag and Au NPs strongly suppress the HIV-1 life cycle before arrival. Furthermore, metallic nanoparticles have antiviral properties against retroviruses [118].

The antivirus pathway of magnesium nanoparticles based on their metal ions, size, and shape—especially stabilized magnesium nanoparticles—reveals a notably higher number of connections, including those between host cells and viruses differentiated with nanoparticles. The antivirus process for MNPs can take place inside or outside of the host cell [119]. The mechanism, which began when nanoparticles communicated with gp120 proteins, terminated host cell coupling positions and suppressed virus adhesion to host cells (Figure 4). Another feasible mechanism was mediated by scattering the virus pieces before they made their way into the cell, which caused viral genome coupling to the virus pieces. The MONPs might be applied as antivirus agents by coupling the materials to the exterior of the virus [120]. This holds the connections between the coupling area on the exterior of the virus and the receivers on the exterior of the host cell. Furthermore, the virus does not enter the cell. MNPs, particularly silver and gold nanoparticles, are said to have antiviral properties against various viruses. Gold nanoparticles terminate the gp120 coupling to CD4 and suppress the virus’s arrival, while silver nanoparticles suppress viral arrival, coupling, and growth. Silver NPs terminate CD4-associated virion coupling, amalgamation, and pathogenesis by connecting with the viral gp120 in the cell-mediated viruses. In dual-standard viruses, the silver NPs, after connecting with the viral genome, terminate virus growth. Zn nanoparticles disturb viral DNA polymerase activity, resulting in the termination of viral growth. The size of Zn nanoparticles combined with virions may inhibit virus entry into the cell. 

### 4.5. Anti-Inflammatory Activity

Anti-inflammatory therapy is a cascade method that induces immune reactive composites such as cytokinins and interleukins that may develop keratinocytes as well as T, B, and C lymphocytes and macrophages. The endocrine system produces several anti-inflammatory mediators, such as antibiotics and enzymes. Another significant anti-inflammatory substance, such as cytokines (IL-1 and IL-2), is formed via the primary immune organs. These anti-inflammatory agents stimulate healing activity. Inflammatory arbitrators occur in biochemical mechanisms and control disease spread. Bio-mediated, obtained AgNPs attain a better wound healing pathway and tissue regrowth in inflammatory features. According to one study, bio-mediated Au and Pt NPs are substitute agents for treating inflammation via the traditional route [121].

In recent years, nanoparticles have been developed as anti-inflammatory agents. Nanoparticles have a high exterior-position-to-interior ratio and are used to block inflammatory agents such as cytokines and inflammation-mediating enzymes, as well as other supplements. Several metal-mediated nanoparticles, such as those based on Ag, Au, copper, and iron oxide, have been reported to have effective anti-inflammatory properties. Swelling is the body’s immediate response to internal breakage, transmission, hormone checks, and damage in the internal shape and outer functions, such as infection by infectious microorganisms or an external component. Individual resistance cells trigger antigen receptors to assume biochemical actions. Inflammation is affected by tissue and cellular damage, which leads to a disparity in the signals managing the inflammation. When injured or infected, tissue produces an inflammatory response that results in the formation of macrophages and killer cells. Macrophages have an important role in auto-inflammatory reactions. Macrophages are large, single-nucleated phagocytes that develop in the bone marrow as completed white blood cells migrate to monocytes in the bloodstream. These monocytes then float to various tissues and develop into macrophages. Macrophages are divided into two steps: pro-inflammatory M1 macrophages, whose development induces inflammation, and anti-inflammatory M2 macrophages, which are activated as an anti-inflammatory reaction and induce the reassembly of the inflamed tissue and organs. Macrophages are capable of withstanding the inflammation reaction by stimulating the two characteristics unexpected in the retarder’s disorder. The macrophages overcome the inflammation and skin injury via phagocytosis, resulting in inflammation via initiation signals inducing the macrophages [122].

### 4.6. Antidiabetic Activity

A type of metabolic disorder in which the blood sugar ratio is uncontrolled is diabetes mellitus. Some foods and stability diets, as well as synthetic drugs, may suppress diabetes in some food scales, making DM therapy a difficult task. Furthermore, the biosynthesized NPs could be used as a replacement drug to treat diabetes mellitus. According to Daisy and Saipriya (2012), Au NPs have better medicinal activity for diabetic control. Au nanoparticles effectively lower the ratio of liver enzymes such as alanine movement, serum creatinine, uric acid alkaline, and phosphatase in exposed diabetes mice. Au NPs exposed to a diabetic model demonstrated a reduction in the HbA (glycosylated hemoglobin) scale that was managing the standard scale [123]. Swarnalatha et al.’s (2012) research stated that *Sphaeranthus amaranthoides*-bio-medially obtained AgNPs suppressed a-amylase and a carbohydrate sugar in diabetes under an animal study [124]. *Premna herbacea Roxb* extract contains antidiabetic properties [125]. Pickup et al. (2008) stated that NPs are important treatment substances for better diabetes management. The medicinal research in mice completely managed the sugar ratio of 140 mg/dL in Ag NP treatment [126].

### 4.7. Antioxidant Activity

Antioxidant activity, along with non-enzymatic and enzymatic agents, manages free radical development. Along with brain injury, atherosclerosis, and cancer, free radicals target cellular breakage. Free radicals are developed through ROS such as hydrogen peroxide and SOD. Bio-constituents such as glycoproteins, proteins, phenolics, lipids, and flavonoids effectively manage free radical development. Furthermore, the scavenging action of antioxidants is essential to controlling several diseases, such as neurodegenerative diseases and metabolic diseases. Silver nanoparticles have stronger antioxidant properties compared to standard drugs such as ascorbic acid.

The nanoparticles revealed increased antioxidant activity, and the tea extract showed increased flavonoids and phenolic components. Yazdi et al. (2020), stated that reactive oxygen species and free radicals have revealed activity in the biological system [127]. These agents are natural metabolic outcomes that damage cell development, which leads to cell breakage, the unreliability of biological components, and damage to common features in several cells.

Oxidative stress is involved in the epidemic of several diseases, including cancer, Alzheimer’s disease, and blindness. Plants have a lot of antioxidants, which protect human health because they strongly preserve biological systems against these substances, select toxic free radicals, and lower cell destruction. The nanomaterial is familiar as a vehicle system for selected drug transmission in current years [128]. The CeO_2_ nanoparticles used as vehicles for cancer therapy due to their antioxidant activities might standardize ROS. Current research states that these nanoparticles have anti-cancer activities during their antioxidant activities and secure healthy cells [129]. Bio-mediated synthesized nanoparticles revealed various biomedical applications, which are depicted in Table 6.

### 4.8. Anticancer Therapy

Cancer is one of the deadliest causes of death today [150]. Cancer develops due to environmental and genetic factors. Several advancements due to the use of different nanoparticles have been made in recent years. Some of the nanoparticles are highlighted in this section. A silver nanoparticle obtained from the probiotic bacteria *L. rhamnosus* GG was very helpful in dealing with HT-29 cells in colorectal cancer [150]. Gold nanoparticles from *L. kimchicus* DCY51T showed excellent anticancer activity against A549 cells (a human lung adenocarcinoma cell line) and HT29 cells (a human colorectal adenocarcinoma cell line) [150]. SeNPs from *L. casei* were very effective against colon cancer cells. CuO NPs obtained from *L. casei* subsp. casei were found to be effective against human gastric carcinoma cells (AGS) and the human colon carcinoma cell line (HT-29) [150]. Pt nanoparticles obtained from *Streptomyces* sp. were found to be effective against the breast cancer MCF-7 cell line [150]. Furthermore, ZnO obtained from *Lactobacillus* spp. was found to be effective against human colon cancer (HT-29) [150]. Thus, it is clear that different nanoparticles obtained from the biosynthesis approach have effective actions toward cancer treatment.

### 4.9. Bio-Sensing Applications

The sensing of biological materials using nanoparticles is very useful for the betterment of mankind [151]. Several nanoparticles are used for these kinds of bio-sensing applications [152]. In a study, chloroplast-mediated green synthesis of Au-Ag alloy was used to analyze cancer [153]. *S. myriocystum*-mediated PtNPs were used for the detection of allergies and asthma [154]. *Hypnea valencia*-mediated synthesis of AuNPs was used to detect pregnancy in women [155]. Furthermore, *Noctiluca scintillans*-mediated biosynthesis of AgNPs was evaluated to detect mouth gum and oral discharge issues [156].

### 4.10. Other Medical Applications

The wound-healing properties of silver NPs obtained by the extracellular method from *Aspergillus niger* were studied using a rat model for an ablation and thermal wound. The studies reported that silver NPs have effective antimicrobial properties and that nano-Ag can control cytokines that occur during wound restoration. Other medical implementation studies with diabetes, especially the suppression of the enzyme PTP, class PTP1B, Interrupting PTP is a cause of various diseases, such as cancer and metabolic diseases [157]. A quick formation of gold nanoparticles containing guavanoic acid via *Psidium guajava* leaf extract was observed. These were applied in the PTP-1B suppressive study, which revealed the importance of suppressive action with an IC_50_ of 1.14 g/mL^−1^. The biofabrication of gold nanoparticles and their implementation on the glucose biosensor for blood glucose identification were reported by Zheng et al. (2022) [158]. The biosensor is used to determine glucose levels in blood samples, revealing the distribution along with commercial clinical protocols. Other research reported that the green medium mediated the obtaining of gold and silver nanoparticles and was reduced by *Brevibacteriumcasei*. These nanoparticles revealed anti-coagulant properties by suppressing the development of blood clots in the sample that attained blood, including silver nanoparticles [159]. The solidity of Au and Ag nanoparticles in the blood was also confirmed through the treatment of the blood plasma with the materials for 1 day, which did not reveal any considerable lowering in the properties [160].

## 5. Conclusions and Future Scope

The nanotechnology field is mainly interconnected with physics, chemistry, biology, and material science, and it creates new biomedical nano-sized particles for therapeutic and pharmaceutical implementations. In recent years, nanoscience has gained researchers’ interest because of its significant applications in medical diagnosis, pharmacy, disease curing, electronics, agriculture, space, and chemical industries. Nanoparticles are currently thought to be extremely useful materials. The biologically mediated nanoparticles are procured through various organisms such as plants, bacteria, fungi, actinomycetes, and yeast. Bio-mediated nanoparticles have been widely used to treat various pathogenic diseases while having fewer toxic effects. Non-biological approaches such as physical and chemical methods are used in the production of nanoparticles, which have a variety of toxic properties for the environment and human health. Hence, as a result, biologically mediated metallic nanoparticles are non-toxic, less expensive, and less harmful to the environment. Moreover, the obtained nanoparticles might stimulate activity using genetic engineering methods. Bio-mediated nanoparticles have specific properties such as being more biocompatible, having a larger surface area, being more reactive, and being non-toxic. The current review provides a clear point of view on various sources of synthesized nanoparticles and their medical applications, such as antibacterial, antifungal, antiviral, anti-inflammatory, antidiabetic, antioxidant, and other medical applications.

## Figures and Tables

**Table 1:** Biosynthesis of nanoparticles using plant extract.

Plant Name	Type of Nanoparticles	Size of Nanoparticles	Biological Activities	Reference
*Desmodium* *triflorum.*	Silver	5–20 nm	Antimicrobial	[46]
*Chrysophyllum* *oliviforme*	Silver	20–50 nm	Antioxidant Anticancer	[47]
*Veronica amygdalina*	Silver	2–18 nm	Antibacterial	[48]
*Cinnamon* *zeylanicum*	Silver	8–12 nm	Antibacterial	[49]
*Phyllanthus amarus*	Silver and Gold	25 & 50 nm	Antibacterial	[50]
*Chrysopogon* *zizanioides*	Silver and Gold	20 & 50 nm	Antibacterial, Antioxidant	[51]
*Camellia sinensis*	Palladium	5–20 nm	Catalytic	[52]
*Green tea*	Iron	50–80 nm	Removal of Hexavalent Chromium	[53]
*Ocimum sanctum*	Silver	10–17 nm	Antibacterial	[54]
*Amaranthus* *spinosus*	Silver	10–50 nm	Antibacterial	[55]
*Cycas pschannae*	ZnO NRs	50–100 nm	Antibacterial	[56]
*Cyrtrandroemia nicobarica*	ZnO NRs	20–200 nm	Antioxidant	[57]
*Knema andamanica*	ZnO NRs	20–200 nm	Antibacterial	[58]
*Leea asiatica*	ZnO NRs	20–200 nm	Antioxidant	[59]
*Leea grandifolia*	ZnO NRs	50–100 nm	Antibacterial	[60]
*Manilkara littoralis*	ZnO NRs	50–100 nm	Antioxidant	[61]

**Table 2:** Biosynthesis of nanoparticles using marine algae.

Marine Algae	Type of Nanoparticles	Size of Nanoparticles	Biological Activities	Reference
*Sargassum wightii*	Gold	8–12 nm	Antibacterial	[62]
*Sargassum wightii*	Silver	6.20 nm	Fabric	[63]
*Ulva fasciata*	Silver	4–10 nm	Antifungal	[64]
*Cystophora moniliforms*	Silver	27–35 nm	Antibacterial	[65]
*Caulerpa racemosa*	Silver	5–25 nm	Antibacterial	[66]

**Table 3:** Biosynthesis of nanoparticles using bacteria.

Bacteria Species	Type of Nanoparticles	Size of Nanoparticles	Biological Activities	Reference
*Lactobacillus plantarum*	Zinc oxide	7 nm	Wound healing	[70]
*Pseudomonas* *Fluorescens*	Silver	20–30 nm	Antibacterial	[71]
*Escherichia coli*	Silver	35 nm	Antibacterial	[72]
*Lactobacillus plantarum*	Silver	4.7–24.3 nm	Antibacterial and Antioxidant Activity	[73]
*Streptomyces* sp	Silver	5–3.9 nm.	Antiparasitic activity	[74]
*Bacillus subtilis*	Titanium dioxide	66–77 nm	Antibacterial	[75]
*Rhodopseudomonas* *Capsulate*	Gold	50–60 nm	Bioreduction	[69]
*Arthrobacter* *nitroguajacolicus*	Gold	40 nm	Antibacterial	[76]
*Lactobacillus* *Fermentum,*	Iron Oxide	10–15 nm	Antibacterial	[77]
*Bacillus* sp.	Silver	5–15 nm	Antibacterial	[78]

**Table 4:** Biosynthesis of nanoparticles using fungi.

Fungi Name and Species	Type of Nanoparticles	Size of Nanoparticles	Biological Activities	Reference
*Aspergillus* *foetidus*	Silver	20–40 nm	Antifungal	[81]
*Aspergillus* sps	Iron	50–200 nm	Iron absorption	[82]
*Aspergillus* sps	Silver	30–210 nm	Antiviral	[83]
*Calophyllum apetalum*	Silver chloride	100 nm	Treating rheumatism and leprosy	[84]
*Aspergillus Niger*	Silver	20 nm	Antibacterial	[85]
*Aspergillus* sp	Silver	3–40 nm	Antibacterial and Anticancer	[86]
*Glycosmis* *mauritiana*	Silver	65 nm	antioxidant, antimicrobial, anti-inflammatory and tyrokinase inhibitory activity	[87]
*Chrysosporium tropicum*	Silver	20–50 nm	Drug formation and diseases diagnosis	[79]
*Fusarium* *oxysporum*	Silver	20–50 nm	Drug formation and diseases diagnosis	[80]
*Penicillium* sp.	Gold	50 nm	Extracellular synthesis	[88]

**Table 6:** Various source-synthesized nanoparticles used for biomedical application.

Source	Source Name	Nanoparticles	Biological Activity	References
Plant	*Cissus arnotiana*	Cu	Antimicrobial and antioxidant properties	[130]
	*Taraxacum laevigatum*	Pt	Antimicrobial activity	[130]
	*Filicium decipiens*	Pd	Antimicrobial activity	[130]
	*Elettaria Cardamomum*	Au	Antimicrobial activity	[130]
	*Trigonella foenum-graecum*	TiO_2_	Antimicrobial activity	[130]
	*Chaenomeles* sp	Fe_2_O_3_	Antibacterial activity	[131]
	*Azardirachta indica* *Coccinia grandis*	CaNPs	Antibacterial activity	[132]
	*Hydrangea paniculata*	Mg and Ag	Health care application	[133]
	*Allamanda cathartica*	AgNPs	Antioxidant and Antibacterial activity	[134]
	*Hylotelephium telephium*	CuO and ZnO	Antioxidant and Antibacterial activity	[135]
Bacteria	*Bacillus cereus*	Ag	Antibacterial activity	[136]
	*Alteromonas macleodii*	Ag	Antibacterial activity	[137]
	*Deinococcus radiodurans*	Ag	Antibacterial activity, anti-biofouling agent and anticancer activity	[138]
	*Pseudomonas aeruginosa*	Ag	Antibacterial activity	[139]
	*Bacillus brevis*	Ag	Antibacterial activity against multi-drug resistant bacteria	[140]
	*Azotobacter vinelandii*	Ag	Antioxidant and Antibacterial activity	[141]
	*Nitrobacter* sp	Ag_2_O	Antioxidant and Antibacterial activity	[142]
Fungi	*Penicillium diversum*	Ag	Antimicrobial activity	[143]
	*Aspergillus foetidus*	Ag	Antifungal activity	[126]
	*Pleurotus ostreatus*	Au	Antimicrobial activity	[144]
Algae	*Ulva lactuca*	Ag	Antiplasmodial activity	[145]
	*Chlorella vulgaris*	Au	Anti-pathogenic activity	[146]
	*Galaxaura elongata*	Ag	Antibacterial activity	[147]
	*Padina tetrastromatica*	Au	Antibacterial activity	[148]
	*Sargassum muticum*	ZnO	Anti-angiogenesis and antiapoptotic activity	[149]

## Data Availability

Not applicable.
